# Lessons from a Health Policy and Systems Research programme exploring the quality and coverage of newborn care in Kenya

**DOI:** 10.1136/bmjgh-2019-001937

**Published:** 2020-01-31

**Authors:** Mike English, David Gathara, Jacinta Nzinga, Pratap Kumar, Fred Were, Osman Warfa, Edna Tallam-Kimaiyo, Mary Nandili, Alfred Obengo, Nancy Abuya, Debra Jackson, Sharon Brownie, Sassy Molyneux, Caroline Olivia Holmes Jones, Georgina A V Murphy, Jacob McKnight

**Affiliations:** 1 Health Services Unit, KEMRI – Wellcome Trust Research Programme, Nairobi, Kenya; 2 Centre for Tropical Medicine & Global Health, Nuffield Department of Clinical Medicine, Oxford, Oxfordshire, UK; 3 Institute of Healthcare Management, Strathmore University Strathmore Business School, Nairobi, Nairobi Area, Kenya; 4 Health-E-Net Limited, Nairobi, Kenya; 5 Department of Paediatrics, University of Nairobi, Nairobi, Nairobi, Kenya; 6 Neonatal, Child and Adolescent Health Unit, Kenya Ministry of Health, Nairobi, Kenya; 7 Nursing Council of Kenya, Nairobi, Kenya; 8 National Nurses Association of Kenya, Nairobi, Kenya; 9 Nairobi City County, Nairobi, Kenya; 10 University of Technology Sydney, Sydney, New South Wales, Australia; 11 Griffith University Menzies Health Institute Queensland, Nathan, Queensland, Australia; 12 Health Systems and Research Ethics, KEMRI-Wellcome Trust Research Programme, Kilifi, Kenya; 13 Centre for Tropical Medicine & Global Health, Nuffield Department of Medicine, Oxford, UK; 14 Center for Tropical Medicine and Global Health, University of Oxford Centre for Tropical Medicine, Oxford, UK; 15 Department of Health System and Research Ethics, KEMRI Wellcome Trust Research Programme, Kilifi, Kenya; 16 Nuffield Department of Medicine, University of Oxford, Oxford, UK

**Keywords:** child health, epidemiology, health services research, health systems evaluation, paediatrics

## Abstract

There are global calls for research to support health system strengthening in low-income and middle-income countries (LMICs). To examine the nature and magnitude of gaps in access and quality of inpatient neonatal care provided to a largely poor urban population, we combined multiple epidemiological and health services methodologies. Conducting this work and generating findings was made possible through extensive formal and informal stakeholder engagement linked to flexibility in the research approach while keeping overall goals in mind. We learnt that 45% of sick newborns requiring hospital care in Nairobi probably do not access a suitable facility and that public hospitals provide 70% of care accessed with private sector care either poor quality or very expensive. Direct observations of care and ethnographic work show that critical nursing workforce shortages prevent delivery of high-quality care in high volume, low-cost facilities and likely threaten patient safety and nurses’ well-being. In these challenging settings, routines and norms have evolved as collective coping strategies so health professionals maintain some sense of achievement in the face of impossible demands. Thus, the health system sustains a functional veneer that belies the stresses undermining quality, compassionate care. No one intervention will dramatically reduce neonatal mortality in this urban setting. In the short term, a substantial increase in the number of health workers, especially nurses, is required. This must be combined with longer term investment to address coverage gaps through redesign of services around functional tiers with improved information systems that support effective governance of public, private and not-for-profit sectors.

**Summary box**

- Multiple research approaches were used to develop an understanding of the context, coverage and quality of inpatient care for sick newborns in Nairobi, Kenya, over a 4-year period; here we try to draw lessons from this body of work.
- Conducting this type of work and generating locally credible findings was only possible through extensive formal and informal stakeholder engagement spanning a diverse set of institutions and perspectives linked with an ability to adapt our research designs as findings emerged while keeping overall goals in mind.
- We found in a predominantly poor, urban population that when hospital care is needed for a sick newborn an estimated 45% cases do not access any suitable facility, that the public sector provides 70% of existing care and that private sector care is either very expensive or offers lower quality care through low-volume facilities.
- In the public and low-cost not-for-profit sectors, critical nursing workforce shortages significantly undermine quality of care, patient safety and nurses’ well-being. The provision of high-quality care is almost impossible in these settings but working routines and norms have emerged that enable professionals to cope and that sustain the appearance of adequate care.
- In the short term, a substantial increase in the number of health workers, especially nurses, is required to improve quality. To improve coverage and quality, this must be combined with longer term investment to redesign systems of care and improve information that supports effective governance.

## Introduction

An influential series of papers at the start of this decade articulated the case for more and better health policy and systems research (HPSR) in low-income and middle-income countries (LMICs).^1–3^ They pointed to the need to consider health systems hardware, software and sociopolitical context as well as the multilevel nature of health systems while reflecting that ‘HPSR has taken form from, and continues to be shaped by, questions bubbling up from the field’ that may be asked by those ‘impelled to resolve practical concerns of service delivery’. The same authors emphasised that HPSR benefits from bringing together positivist and relativist approaches to deepen understanding of how to intervene within existing health systems where ‘Health policies and systems are fundamentally shaped by political decision-making, [and] the routines of health systems are brought alive through the relationships among the actors involved in managing, delivering, and accessing healthcare’.^1^ HPSR also then benefits from being embedded in a context and close engagement with local actors.^2^

Here we focus on what was learnt from a 4-year programme of HPSR work on newborn hospital care in Nairobi, Kenya. It aimed to take up the challenges of being embedded in context, engaging closely with local actors and learning from different forms of enquiry. The research was prompted by informal observation of problems with service delivery at multiple health system levels.^4 5^ Nursing workforce shortages were a concern and so the programme sought to explore its consequences and the potential for task-sharing as a partial solution while recognising its challenges.^6 7^ This prompted research to understand the day-to-day work routines on neonatal wards and the perspectives of different stakeholder groups.^3^

### Why focus on newborn care as a service delivery problem?

Neonatal mortality now accounts for 45% of all child mortality in Kenya and elsewhere, and specific targets have been set as part of the Sustainable Development Goals to reduce it to ≤12/1000 live births (from 22/1000 in Kenya).^8^ Among a set of key strategies to achieve this target is provision of basic, high-quality inpatient care.^9^ In multicountry studies, the most significant bottlenecks to providing this care are with the health workforce and service delivery.^10^

The delivery of high-quality hospital care for sick newborns poses multiple challenges. At population level, good outcomes depend on access to appropriately resourced service delivery units that can sustain effective, safe and respectful care comprising multiple interventions over many days.^11^ With neonatal mortality rates in Nairobi City County estimated to be 39/1000 live births,^12^ the highest in Kenya, it seemed health systems were failing to deliver effective services.

### Context

In 2013, health service delivery was devolved from national to county levels in Kenya with the new Nairobi City County government responsible for approximately 4.5 million people. In this urban population, substantial proportions are food-poor and hard-core poor.^13 14^ The public sector aims to provide the whole population with free facility-based delivery and newborn care (although limited copayments may still be common).^15^ Being a capital city, both private sector and not-for-profit (often faith-based) providers are common, but there was little understanding of how all sectors combined to provide inpatient newborn care, a knowledge gap we aimed to address.

The human resources for health crisis affecting LMIC is well recognised.^16 17^ In higher income countries, a growing body of knowledge demonstrates the importance of nurses to patient safety, outcomes and care quality.^18 19^ In LMIC, however, nurses’ contribution to inpatient quality of care outside maternity units has rarely been examined. We aimed to address this gap and explore whether some simpler tasks currently carried out by professional nurses on inpatient newborn units (NBUs) might be delegated to others. Although healthcare assistants (HCAs) in high-income countries now provide much basic patient care,^6^ there is no policy or regulatory framework supporting such a cadre in Kenya. HCAs are not a replacement for professional nurses, but they may offer a means to support the professional workforce in contextually appropriate skill-mix models. Theoretically, HCA might then support cost-efficient improvement in coverage and quality. However, task shifting/sharing is not a simple technical solution. New cadres may be rejected by existing professions, may exacerbate existing structural challenges and improvements in quality and safety cannot be guaranteed.^6 7 20 21^

### Guiding principles

**Figure 1:**
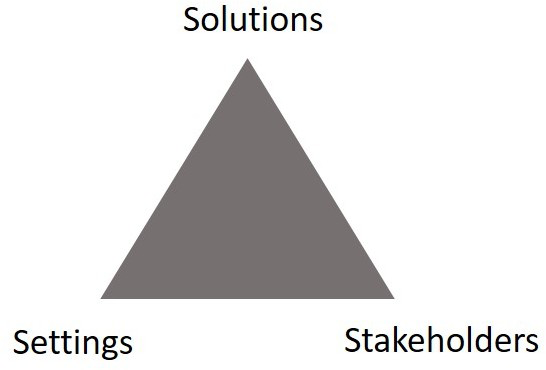
Guiding the research team’s work were key principles that to identify appropriate solutions a deep understanding of the setting or context and the stakeholders who shape the health system are required.

**Figure 2:**
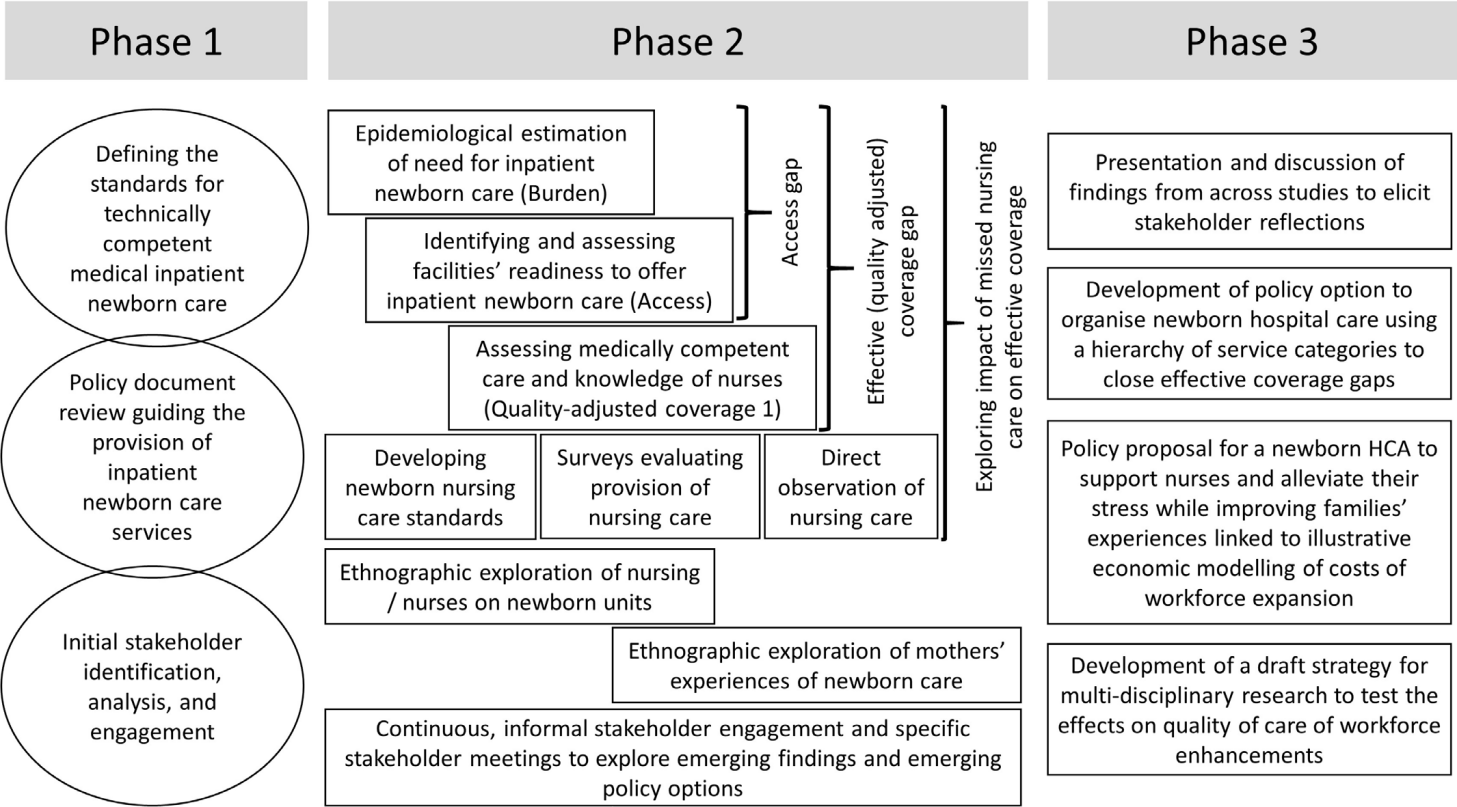
Schematic representation of the research and engagement activities conducted as part of the Health Services that Deliver for Newborns (HSD-N) Programme. The HSD-N Programme was designed and implemented in three phases for simplicity illustrated as distinct but, in reality, overlapping. Of these, phase two was the longest. Throughout researchers from different disciplinary backgrounds interacted and where appropriate collaborated. A major thrust of the quantitative research was to combine evaluations to develop a better understanding of how likely it was that a sick newborn would receive quality inpatient care in Nairobi. This was complemented by detailed qualitative work that explored the reality of newborn ward hospital care, how nurses in particular coped with and families experienced such a high-pressure environment. All forms of data informed stakeholder discussions and development of draft policy proposals. HCA, healthcare assistant.

To understand challenges and identify potential solutions engagement with local stakeholders was felt to be key. This included senior-level and midlevel figures in the national and county ministries of health, senior personnel from medical and nursing regulatory bodies, professional associations, training institutions and staff from public, private and not-for-profit hospitals. We found that this engagement often had to be opportunistic, informal and wide ranging, a process that was facilitated by our research team being embedded in the context and able to undertake engagement in the form of a continuous ‘conversation’ (figure 1). Where definitive outputs based on consensus were needed, more formal stakeholder engagement workshops were necessary, and these were regular features of our overall programme of work (figure 2). Engagement therefore takes time for all concerned, requires investments in building relationships and cannot be handed off to a communications team.

We now focus on specific lessons learnt before reflecting on broader lessons and then discussing their implications.

## Access, readiness and technical quality of neonatal hospital care in Nairobi City County

Without comprehensive and high-quality vital statistics on all births, deaths and their cause, and without well-functioning health information systems that capture all severe neonatal illness episodes across sectors, the need for inpatient neonatal care must be estimated to evaluate access.^22^ Local stakeholders can then help identify all facilities meeting threshold criteria of providing inpatient neonatal care 24 hours a day for 7 days a week. To begin to understand quality-adjusted access means assessing the coverage and quality provided by these facilities. We used detailed surveys to determine the number of sick newborns reaching facilities and to evaluate quality in terms of infrastructural readiness, the technical quality of medical care^23^ and nursing knowledge^24^ all compared with care standards codified as guidelines.^25^ Combining all these results enabled us to estimate: (1) the proportion of sick newborns likely to require inpatient care in a typical year that accessed a facility ready to offer high-quality essential inpatient neonatal care and (2) the likelihood of receiving competent medical care and care from nurses having adequate levels of knowledge. Based on these data together with estimates of the population in need, we inferred that 45% of sick newborns were not accessing any suitable facility while basic quality-adjusted *effective coverage* with inpatient newborn care was only 24%.^26^

## Deeper exploration of the quality of care: a focus on nursing

Most quality assessments in LMIC hospitals pay little attention to the ongoing process of inpatient nursing care. In Kenya, we began this process by working with stakeholders to draft minimum standards for nursing care on NBU.^27^ This involved characterising key nursing tasks and developing consensus on how frequently they should be performed and by whom.^27^ Based on nurses’ self-reports, we learnt that even nurses with correct knowledge are often not able to provide appropriate care, with multiple care tasks missed and many already being shared informally with unskilled hospital staff and mothers. Most commonly this was attributed to the high numbers of babies each nurse was caring for.^28^

Self-reports may, however, be subject to bias. We felt we had to deepen our enquiry and so proceeded to make direct observations of whether nursing care was missed in selected Nairobi facilities.^29^ Working with stakeholders to agree standards and design tools enabled us to provide credible reports of striking levels of ‘missed nursing care’ strongly associated with periods when a nurse looked after large numbers of babies.^30^ These data are important as four public hospitals and one low-cost, not-for-profit hospital together provide over 80% of all inpatient newborn care in Nairobi City County.^23^ It is these hospitals that have fewest staff (often >12 babies being cared for by each nurse) and cater to Nairobi’s large population of urban poor in often overcrowded wards.^23^

**Figure 3:**
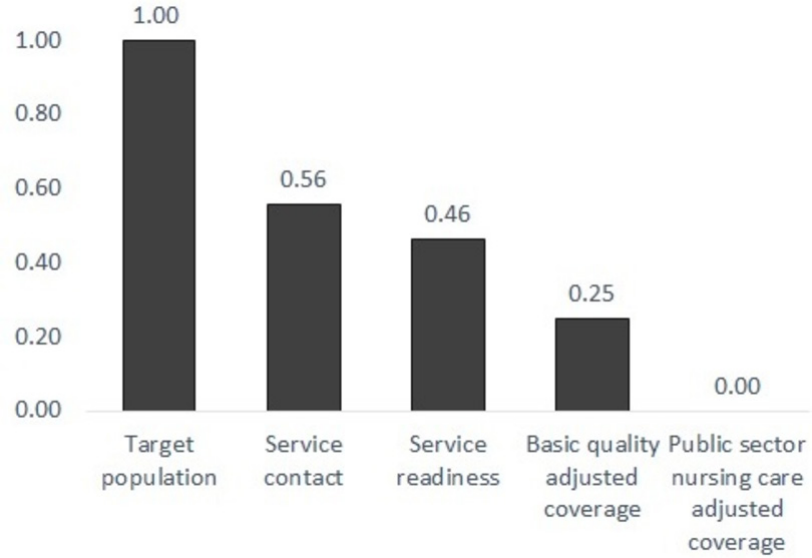
Illustrating the likely cascade of coverage with and quality of essential inpatient care for sick newborns in Nairobi City County. With a target population of sick newborns likely to need inpatient care of 21 966 in the year 2018, we used multiple data sources to estimate proportions (x-axis) accessing care represented as an ordered cascade of progressively more stringent quality criteria. In this approach, probability estimates from different studies are multiplied as additional quality criteria are introduced to provide an indication of the points, or steps, in the cascade where coverage and quality gaps have most impact on the reference population (the initial bar). The criteria we sequentially introduce are: (1) service contact: the proportion reaching a facility potentially capable of offering such care based on our survey of all neonatal unit admissions in a year; (2) service readiness: the proportion of those accessing care that reach a facility that has a minimum set of resources to support essential care; (3) basic quality-adjusted coverage: the proportion reaching a facility with needed resources who are then likely to receive technically competent medical care; and (4) public sector nursing care adjusted coverage, which further adjusts for the likelihood that nurses in public sector hospitals that provide >70% of inpatient newborn care will be able to complete 80% or more of tasks comprising a minimum standard of nursing care. (Estimated probabilities used in constructing the cascade are derived from previously published work.^23 26 30^).

When we combine these new forms of nursing quality assessment to develop an overall picture of coverage and quality, we see a major challenge in access to a suitable facility by the population in need with coverage that is heavily dependent on the public sector. As public hospitals largely meet basic readiness criteria, adding care competence as an additional criterion results in a more marked step-down in the neonatal inpatient care quality cascade (figure 3). However, if we focus on the public sector specifically, and adjust for our observations of nursing care delivered, data suggest quality-adjusted effective coverage is close to 0% where one nurse cares for large numbers of sick babies, the situation that predominates in public sector and low-cost, high-volume not-for-profit hospitals (figure 3). We reach this conclusion because the nurses in these facilities typically care for at least 12 babies each, a threshold above which our direct observational data suggest the agreed minimum standard of nursing was not delivered.^30^

## Understanding nurses work

Understanding nurses’ work and why minimum standards may not be met needs a different approach. We used detailed ethnographic work on NBU spanning weekends and nights (work that raised important ethical challenges)^31^ for this purpose. We learnt how nurses’ routines are shaped by long-standing organisational work patterns (such as shifts and the handovers these necessitate), by powerful professional norms (such as the need to complete specific nursing documentation – the ‘Kardex’) and by the practical norms that have evolved in such challenging contexts (such as an acceptance that a nurse working a 13-hour shift alone at night might sleep at the desk).^32^ This approach also helped us see how nurses navigate an apparently overwhelming volume of tasks by conducting a form of subconscious triage to implicitly ration care, typically prioritising delivery of biomedical interventions to the most ill (confirming the findings of quantitative observations).^30^ When work is further complicated by unpredictable ‘crises’, such as resuscitation events, tasks perceived as less critical are missed or delegated with minimal or no supervision to untrained staff or mothers.^32^ It is important to remember, however, that these organisational routines and local practical norms are a response to very difficult working conditions. They enable nurses to maintain some sense of achievement by completing critical tasks; this preserves their identity as capable professionals in the face of considerable adversity.^33^

What this results in is an implicitly accepted lowering of the quality and safety bar (into the ‘illegal normal’)^34^ that helps protect nurses from feeling completely overwhelmed by their impossible situation. These collective strategies help preserve nurses’ dignity so they can cope in severely under-resourced environments, while the health system maintains a functioning veneer. All the while, work everyone agrees represents basic minimum standards such as regular vital signs observations, monitoring intravenous fluid intakes and support for mothers and families is commonly missed.^30 32 33^

## The potential space for task sharing

In the face of impossible workloads, and the toll this takes on staff, the rational and economic case for task-sharing to lower skilled (and lower paid) workers such as HCAs seems clear, suggesting the process could be straightforward. Literature suggests the reality can be rather different.^21 35–37^ Any new cadre in the public sector would be introduced into settings beset by multiple challenges and with deeply embedded working practices, professional norms and jurisdictions. Moreover, our discussions with stakeholders highlight key issues of policy, regulation and professional identity linked to task sharing that go well beyond the localised context of NBU.^38^ Particular concerns around HCA include that they: (1) could be seen by those financing and managing care as (cheap) substitutes for nurses further reducing professionals’ employment possibilities, (2) might misrepresent themselves as nurses in the private sector where very small-scale, poorly regulated healthcare outlets have proliferated and (3) might take on bedside tasks that would undermine nurses roles in providing holistic care, a central part of their professional identity.^38^

We learnt, however, that while some of these concerns persisted across the 4-year study period that our multiple formal and informal stakeholder engagement activities gave greater prominence to the voices of nurses at the frontline of care who joined consultative panels and workshops. These practising nurses considered introducing HCA to take on less-skilled nursing tasks much more favourably while being clear that increasing nurse staffing is the first priority and that HCA should not be seen as substitutes. Thus, frontline nurses were concerned with alleviating the intense workplace pressure that undermines their relationships with families and results in missed care and with the additional work effectively delegating and supervising HCA might create. As our discussions continued, simulations of how task sharing might be used to support an expansion in the nursing workforce to help achieve coverage and quality goals most efficiently proved an effective and stimulating way to engage with stakeholders.^39^

## Broader learning

**Figure 4:**
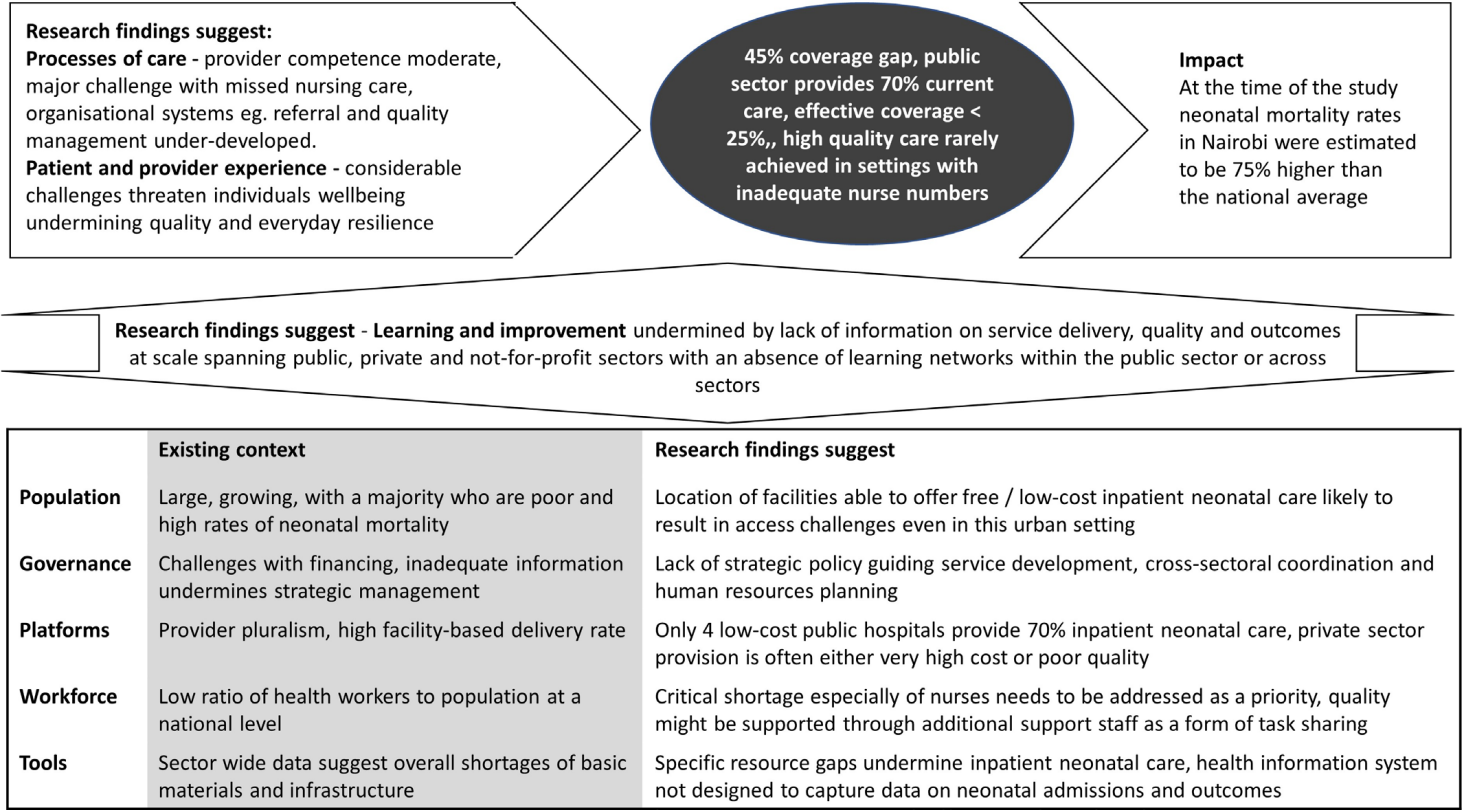
Overview of research programme findings using the High Quality Health Systems in the Sustainable Development Goal Era framework to illustrate that addressing multiple system weaknesses is likely to be necessary to deliver high-quality care at scale and reduce high neonatal mortality.^11^ In this figure, challenges spanning the five platforms at the base of the figure are summarised. These undermine the system’s ability to learn and improve and contribute to inadequacies in the processes of care. All influence the observed outputs of the system (central shaded oval) that are on the pathway to the health impacts the system currently achieves.

We use the framework from the Lancet Global Health Commission on High Quality Health Systems to show how multiple system weaknesses prevent the delivery of high-quality neonatal inpatient care at scale (figure 4).^11^ Our results indicate clearly that no one intervention, innovation or technology is likely to reduce Nairobi’s high neonatal mortality. Broader health system strengthening is needed as part of a longer term agenda if effective coverage with key services is to be achieved. Our work highlights four interlinked areas.

1. Health information: effective planning and governance demand that accurate information on the nature and distribution of resources, facility utilisation and patterns of serious morbidity and mortality (and more)^40^ are readily available. Without such data, we risk the ‘scandal of invisibility’,^41^ especially for the poor, and it should not require researchers to identify which facilities provide which services and at what volumes. These data must span all sectors. Our assessments suggested some high-cost private sector care was high quality but that quality of care in multiple low-volume private facilities is less good than in the public sector.^23 26^ Improved neonatal inpatient care for the poor should therefore focus on improving care in public and low-cost faith-based facilities.
2. Service delivery needs to be better designed for the long term: there is no detailed strategy to guide investments for inpatient newborn care in Nairobi, instead what we observe is a patchwork of partners supporting specific projects often narrowly focused in scope and geography. We suggest strategic investments are needed in upgrading some facilities to provide standard levels of inpatient neonatal care to decongest the existing public hospitals we studied which should be upgraded to provide an intermediate category of services. Systems that support upward and downward referrals are then needed. This must be linked with similar planning around maternity and other inpatient care services.^42^ Addressing this gap seems critical for a city growing rapidly as Kenya urbanises.
3. We must address nursing workforce shortages in the public and low-cost not-for-profit sectors: it is overwhelmingly clear that nurses cannot provide quality care if each of them is caring for large numbers of sick newborns. This means health spending must increase substantially in line with commitments made a decade ago in Abuja and include specific investments in nursing.^43 44^ Increasing nurse numbers would likely benefit newborns, their families and staff well-being. HCAs are not a substitute for nurses but together with additional nurses might offer a useful skill-mix solution in some settings to improve quality and coverage relatively efficiently.^39^ However, introducing HCA is not a simple technical solution, and studies to explore any impact they have on quality, safety, teamwork and families’ experiences are required as a precursor to any widespread deployment.
4. We need greater capacity for HPSR research that actively engages multiple stakeholders: it is very important to pay attention to context and understand it as dynamic, something our multiple stakeholders continuously reminded us of with their diversity of perspectives. We also need to recognise that there are unlikely to be simple, generalisable solutions. This emphasises the need to increase the capacity for HPSR embedded within LMIC. As in any other field, this will require long-term investments. These might best be mutual endeavours between countries’ governments, research institutions and their international partners, to create what Bennett and colleagues refer to as ‘Institutional Homes’ where ‘cross-boundary competencies’ are fostered.^3^

In this report, we illustrate some of the specific and broader lessons we have learnt over a period of 4 years. A number of reports have been published in keeping with their specific research paradigm. Perhaps a limitation of our work is that we did not construct it with a final form of integrated analysis in mind or with the aim of offering summative explanatory theory.^45–47^ Conducting this set of work was, however, a considerable learning journey for a research team drawn from multiple disciplines and our multiple stakeholders who, we feel, all benefited from experiencing the ‘independent contributions of discrete traditions of enquiry, as well as from the mixing of disciplinary influences’.^1^

## Conclusion

There still seems to be a pervasive notion, reinforced by some global health funding calls, that if we just found the right intervention, innovation or technology, we could dramatically reduce neonatal mortality. Our findings indicate this notion is misguided. We believe that the intervention most likely to improve neonatal hospital outcomes in the short term is a substantial increase in the number of health workers, especially nurses, in facilities that provide free or very low-cost care. This must be combined with longer term investment to redesign services around functional tiers and referral systems to improve coverage and quality together with improved information systems that support effective governance across all sectors.
